# Isolated tuberculous pericarditis, an unusual presentation of tuberculosis – a case report

**DOI:** 10.1186/1471-2334-13-S1-P84

**Published:** 2013-12-16

**Authors:** Raluca Jipa, Mihaela Rădulescu, Adriana Hristea, Ruxandra Moroti, Roxana Petre, Raluca Dulamă, Doina Cristea, Victoria Aramă

**Affiliations:** 1 National Institute for Infectious Diseases “Prof. Dr. Matei Balş”, Bucharest, Romania; 2 Carol Davila University of Medicine and Pharmacy, Bucharest, Romania

## Background

Although during the past years a constant decreasing trend of extrapulmonary tuberculosis (TB) was registered in Romania, the number of reported cases of pericardial effusion TB remained steady at 40-50 cases annually, most of them in association with pleural effusion. Pericardial TB is a potentially lethal complication of TB and it is associated with high rates of morbidity and mortality.

## Case report

We report the case of a 45 year-old man with a known history of psoriasis under biological treatment, diabetes mellitus type 2 and hypertension, admitted for fever and persistent cough for one week. No history of dyspnea, chest pain or hemoptysis was reported by the patient. According to the medical data, prior to the initiation of the biological therapy he had a positive Quantiferon test, and underwent 9 months of isoniazid prophylaxis. The chest X-ray showed an emphasized bilateral basal pulmonary drawing, predominantly on the left side. He has been treated with broad spectrum antibiotics but did not respond well. The echocardiography revealed a large pericardial effusion, with signs of incipient cardiac tamponade. Spiral computed tomography of the chest showed significant pericardial effusion (~3.5 cm in thickness) all around the heart, but no active pulmonary lesions or pleural effusion. Pericardiocentesis was performed and 800 mL of blood stained fluid was drained. The pericardial fluid had a cell count of 3000 cells/cmm, with 95% lymphocytes and normal chemistry. Examination of the pericardial biopsy specimen revealed granulomas. We initiated the anti-TB therapy, with good tolerance.

## Conclusion

TB pericardial effusion remains an uncommon form of extrapulmonary TB, but a high index of suspicion should be kept in mind, especially in immunocompromised patients.

